# Stromal Cells in Early Inflammation-Related Pancreatic Carcinogenesis—Biology and Its Potential Role in Therapeutic Targeting

**DOI:** 10.3390/cancers17091541

**Published:** 2025-05-01

**Authors:** Tina Seidel, Nupur Ohri, Markus Glaß, Yoshiaki Sunami, Lutz P. Müller, Jörg Kleeff

**Affiliations:** 1 Department of Internal Medicine, University Hospital Halle, 06120 Halle (Saale), Germany; tina.seidel@uk-halle.de (T.S.); lutz.mueller@uk-halle.de (L.P.M.); 2 Department of Visceral, Vascular and Endocrine Surgery, University Hospital Halle, 06120 Halle (Saale), Germany; nupur.ohri@uk-halle.de (N.O.); yoshiaki.sunami@uk-halle.de (Y.S.); 3 Institute of Molecular Medicine, Martin Luther University Halle-Wittenberg, 06108 Halle (Saale), Germany; markuss.glass@medizin.uni.halle.de

**Keywords:** pancreatic adenocarcinoma, cancer-associated fibroblasts, pancreatic stellate cells, mesenchymal stem cells

## Abstract

The stroma of healthy pancreases contains various non-hematopoietic, non-endothelial mesenchymal cells. It is altered by chronic inflammation which in turn is a major contributor to the development of pancreatic adenocarcinoma (PDAC). In PDAC, the stroma plays a decisive and well-investigated role for tumor progression and therapy response. This review addresses the central role of stromal cells in the early inflammation-driven development of PDAC. It focuses on major subpopulations of pancreatic mesenchymal cells, i.e., fibroblasts, pancreatic stellate cells, and multipotent stroma cells, particularly their activation and functional alterations upon chronic inflammation including the development of different types of carcinoma-associated fibroblasts. In the second part, the current knowledge on the impact of activated stroma cells on acinar-to-ductal metaplasia and the transition to pancreatic intraepithelial neoplasia is summarized. Finally, putative strategies to target stroma cells and their signaling in early pancreatic carcinogenesis are reflected. In summary, the current data show that the activation of pancreatic stroma cells and the resulting fibrotic changes has pro- and anti-carcinogenetic effects but, overall, creates a carcinogenesis-promoting microenvironment. However, this is a dynamic process and the therapeutic targeting of specific pathways and cells requires in-depth knowledge of the molecular interplay of various cell types.

## Simple Summary

The development of pancreatic cancer is strongly influenced by chronic inflammation, which induces changes in the pancreatic stroma—the connective tissue that includes cell types such as fibroblasts, pancreatic stellate cells, and multipotent stromal cells. While it is well established that the stroma plays a significant role in advanced stages of the disease, its contribution during the early, inflammation-driven phases of tumorigenesis remains poorly understood. This review aims to provide deeper insight into the function and transformation of stromal cells during the initial stages of disease development. Gaining this knowledge could support the development of novel therapeutic strategies to prevent cancer at an early stage and drive forward research on the tumor microenvironment.

## 1. Introduction

In healthy pancreases, the stroma serves as a protective environment that provides essential signals to ensure tissue maintenance and function and suppress malignant transformation [1]. However, persistent inflammation induces changes in the phenotype and function of stromal cells, eventually resulting in fibrosis and contributing to carcinogenesis. This is proven not just by murine models [2] but also by the increased risk for developing pancreatic ductal adenocarcinoma (PDAC) in patients with chronic pancreatitis [3]. The chronic inflammatory process does not only involve epithelial cells of the exocrine system and hematopoietic immune cells but also the non-hematopoietic compartment of mesenchymal cells [4]. In the present review, we summarize the current knowledge regarding the differential types of non-hematopoietic mesenchymal cells present in the pancreas and their inflammation-driven alterations contributing to PDAC carcinogenesis. Based on that, we reflect on targeting the altered pancreatic stroma as an approach to prevent inflammation-driven PDAC as well as to provide options for its early treatment.

## 2. Types of Non-Hematopoietic Mesenchymal Cells in Healthy Pancreas

**Figure 1:**
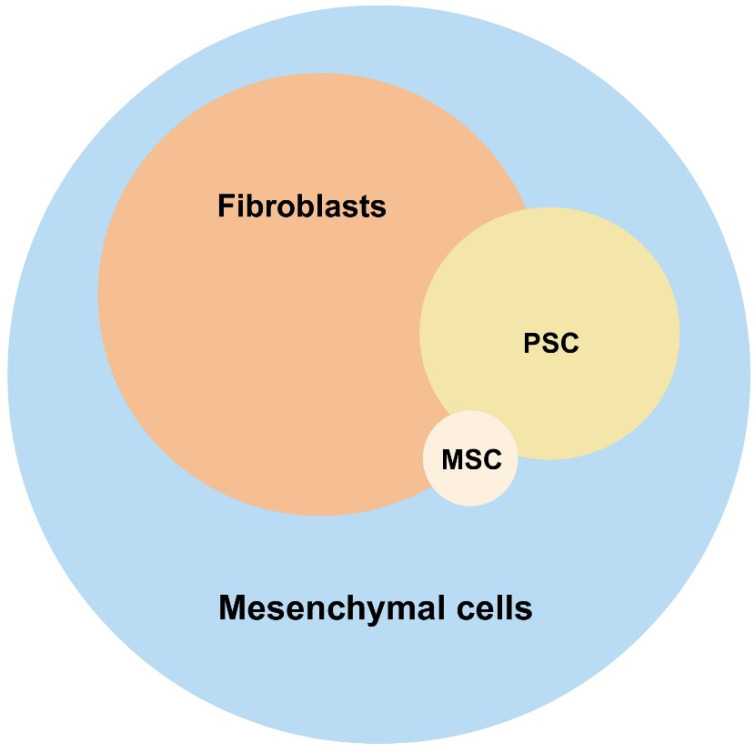
Composition of a healthy pancreatic stroma. The different types of non-hematopoietic stroma cell (fibroblasts, PSCs, and MSCs) as parts of the mesenchymal cell entity is depicted including the reflection of the size of the respective subpopulation. PSC: pancreatic stellate cells; MSC: multipotent stromal cells. Created in https://BioRender.com.

**Figure 2:**
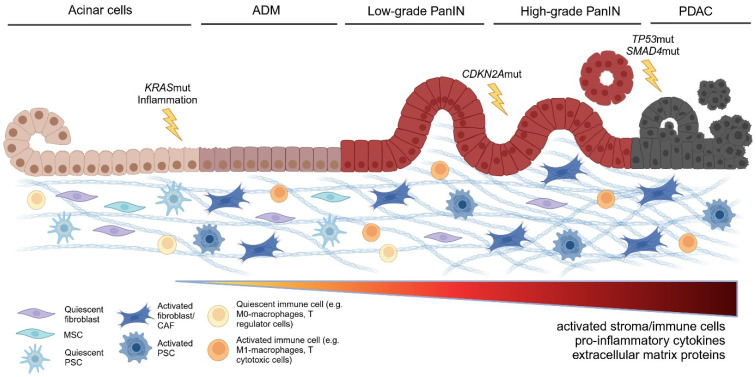
Development of the non-hematopoietic pancreatic stroma during early PDAC carcinogenesis. Depicted is the development of pancreatic adenocarcinoma (PDAC), starting from healthy acinar cells through various precancerous stages to malignant disease including typical mutations and molecular and cellular changes in the microenvironment of the tumor cells. *Acinar cells (healthy pancreas)*: the microenvironment consists of resting fibroblasts, multipotent stromal cells (MSCs), resting pancreatic stellate cells (PSCs), and resting immune cells. *Acinar-to-ductal metaplasia (ADM)*: initiated by activation of KRAS mutations and inflammation. Acinar cells begin to adopt a ductal phenotype. Fibroblasts, MSCs, PSCs, and immune cells are activated, accompanied by immune cell migration and increased extracellular matrix (ECM) production. *Pancreatic intraepithelial neoplasia (PanIN)*: early to advanced precancerous stages with loss of epithelial arrangement and function. The microenvironment shows increased activation of fibroblasts, MSCs, PSCs, and immune cells, as well as increased production of pro-inflammatory cytokines and increasing fibrosis. PDAC: malignant stage with mutations in TP53 and SMAD4. Highly activated tumor microenvironment: pro-inflammatory milieu and pronounced fibrosis. Created in https://BioRender.com.

Fibroblasts, pancreatic stellate cells (PSCs), and multipotent stroma cells (MSCs) have been reported as the major non-hematopoietic, non-endothelial constituents of the stroma in healthy pancreases (Figure 1). However, the distinctions between these cell types are not clearly defined. Thus, it remains questionable whether these cell types uniquely exist in vivo or rather represent different stages of a continuous differentiation process of mesenchymal cells. Still, the existing data and literature justify a distinction between these cell types as they harbor distinct functional characteristics. Based on current data, they appear to coexist in healthy pancreases in a resident, non-activated state (Figure 2).

### 2.1. Tissue-Resident Fibroblasts in Pancreas—Origin and Markers

Fibroblasts are mesenchymal cells [5], and pancreatic tissue-resident fibroblasts originate from Insulin Gene Enhancer Protein ISL-1-expressing splanchnic mesenchyme during fetal development [6]. Lineage tracing studies in mice have shown that the splanchnic mesenchyme, the fetal cell layer surrounding the endoderm from which the pancreatic epithelium develops, gives rise to the majority of resident pancreatic fibroblasts in the healthy pancreas [6]. These fibroblasts are localized to perivascular regions and the tissue parenchyma, allowing direct interactions with epithelial cells [7]. In general, fibroblasts are spindle-shaped, non-epithelial, non-immune, and non-hematopoietic cells that typically exist in a quiescent or inactive state, with minimal metabolic and transcriptomic activity [8]. Upon activation by factors such as transforming growth factor-β (TGF-β), platelet-derived growth factor (PDGF), or interleukin-6 (IL-6 quiescent fibroblasts transdifferentiate into an activated, myofibroblast-like phenotype [9,10]). This activated state is associated with increased proliferation, migration, enhanced production of growth factors, and extracellular matrix (ECM) components. Activated fibroblasts are characterized by the expression of α-smooth muscle actin (α-SMA, encoded by the *ACTA2* gene) [11,12]. The myofibroblastic differentiation plays a role in wound healing [13], acute and chronic inflammation, and fibrosis [12,13]. Fibroblast-specific protein 1 (FSP1) is often used to identify quiescent, non-proliferating (Ki67-negative) fibroblasts [14]. Markers commonly associated with activated fibroblasts include vimentin, αSMA, fibroblast activation protein (FAP), PDGF receptor-α (PDGFRα), PDGFRβ, and discoidin domain-containing receptor 2 (DDR2) [15]. However, the unambiguous characterization of fibroblasts is often complicated due to the lack of specificity of the aforementioned marker proteins. For instance, FSP1 is also expressed by macrophages and certain cancer cells [16] while FAP is found in subsets of CD45^+^ immune cells [17].

Recent lineage tracing experiments have identified two distinct fibroblast populations in the healthy pancreas, distinguished by the expression of transcription factors Gli1 and Hoxb6. Gli1, a key target of the Hedgehog signaling pathway [18], is active during embryonic gastrointestinal development but is suppressed during pancreas formation, with low-level signaling persisting for homeostasis and injury recovery [19]. A subset of healthy pancreatic fibroblasts express Gli1, though they do not resemble pancreatic stellate cells [20]. Hoxb6 is broadly expressed in the mesenchyme of the developing pancreas [21] and remains present in a subset of fibroblasts in the adult pancreas. Currently, no unique markers distinguish pancreatic fibroblasts from fibroblasts in other organs [20]. However, lineage-tracing studies highlight their unique embryonic origins, emphasizing the need for further research to identify distinct pancreatic fibroblast markers and better characterize their role in pancreatic homeostasis and disease.

### 2.2. Pancreatic Stellate Cells—Overlapping and Distinct Characteristics Compared to Pancreatic Fibroblasts

Pancreatic stellate cells (PSCs) are mesenchymal cells with a star-shaped morphology that constitute approximately 7% of the cellular population in the pancreas [22]. They are primarily located around small pancreatic ducts and blood vessels, and in the basolateral regions of pancreatic acinar cells [23]. The developmental origin of PSCs remains a topic of debate, with evidence suggesting contributions from mesenchymal [22,24], endodermal [25], and neuroectodermal lineages [26]. Initially described by Watari in 1982, PSCs were noted for their periacinar localization and their resemblance to hepatic stellate cells. The isolation and culture from rat and human pancreas were optimized in 1998 [23,27] enabling extensive in vitro studies of PSC biology.

In a healthy pancreas, PSCs exist in a quiescent state, characterized by the presence of vitamin A-rich lipid droplets in cytoplasm. The physiological role of vitamin A storage in PSCs remains unclear, but it is hypothesized to contribute to maintaining quiescence [28]. Upon activation in response to injury or inflammation, PSCs lose their lipid droplets presumably due to use of the stored lipids for energy supply and membrane structures [29,30,31]. This activation is associated with increased proliferation, migration, ECM production, and expression of markers such as α-SMA, FAP, PDGFRα/β, collagens (I and III), fibronectin, ICAM-1, cadherin-11, DDR2, vimentin, GFAP, NGF, NCAM, desmin, nestin, etc. [8,23,28,32,33,34].

PSCs and pancreatic fibroblasts share similarities, but they also exhibit key differences in their phenotype and biology. Both are of mesenchymal origin and can activate into a myofibroblast-like phenotype, expressing common markers such as α-SMA, PDGFRα/β, and vimentin [15,28]. Additionally, they share roles in tissue homeostasis, wound healing, and ECM remodeling [35]. Besides their ability to store lipid droplets PSC differ from pancreatic fibroblasts on their expression of GFAP, higher ECM production, and unique expression of scavenger receptors, like CD36, CCK1/2 receptors, and the ACh receptor (Acetylcholine receptor) [36,37]. Traditionally, CCK was thought to stimulate pancreatic enzyme secretion via vagus nerve activation. However, an alternative mechanism involves PSCs. These are positioned basolaterally to acinar cells. Upon CCK stimulation, PSCs release ACh, which then acts on muscarinic receptors on acinar cells to promote digestive enzyme secretions [36]. This suggests that quiescent PSCs may play an unappreciated role in pancreatic exocrine regulation. PSCs express desmin, a marker for stellate cells, with over 90% also positive for α-SMA. In contrast, pancreatic fibroblasts show minimal desmin expression, distinguishing them from PSC markers [38,39]. Moreover, the high α-SMA expression in PSCs enhances their contractile properties, allowing them to regulate vascular and ductal tone in the pancreas. Given their localization around blood vessels and ductal structures, PSC-mediated contraction may influence pancreatic blood flow and secretion [28].

In the healthy pancreas, PSCs and fibroblasts contribute to ECM production and remodeling. This dynamic process is essential for tissue development, homeostasis, and repair following injury [35]. ECM homeostasis relies on the balance between ECM synthesis and degradation, mediated by MMPs [40]. PSCs and pancreatic fibroblasts produce key ECM components, including collagen type I, III, IV, and V, laminins, and fibronectin [41]. Additionally, PSCs regulate ECM turnover by also producing MMPs [42], ensuring matrix remodeling.

Beyond ECM regulation, PSCs also participate in immune regulation. They express cell adhesion molecules, such as ICAM-1, which facilitate immune cell recruitment [43]. Furthermore, PSCs express Toll-like receptors (TLRs), including TLR2, TLR4, TLR3, and TLR5, allowing them to recognize bacterial and viral components and activate innate immune responses [44]. Through the phagocytosis of necrotic debris and aged polymorphonuclear cells [45], PSCs contribute to inflammation resolution, tissue repair, and immune homeostasis in the pancreas.

### 2.3. Multipotent Stroma Cells

Multipotent stroma cells (MSCs)—originally named mesenchymal stem cells—represent a cell type of mesodermal origin and multipotent differentiation potential. According to consensus criteria, the defining minimal characteristics of human MSCs comprise the following: (1) the ability to establish colony-forming unitary fibroblasts (CFU-F) in vitro of spindle-shaped morphologic appearance (similar to fibroblasts); (2) a surface marker profile of CD105^+^, CD73^+^, CD90^+^, CD45^−^, CD34^−^, CD14^−^, CD11b^−^, CD79α^−^, CD19^−^, HLA-DR^−^; (3) the ability to differentiate into osteoblasts, adipocytes, and chondroblasts [46,47]. Obviously, these criteria leave space for much heterogeneity and are not suited to define MSCs in vivo. MSCs have originally been described in the bone marrow but can also be isolated from various other tissues of the adult organism, such as cartilage, skeletal muscle, or adipose tissue [48]. It remains open whether the MSCs from different tissues are distinct or represent a uniform cell population. In general, human tissue-resident MSCs are impacted by age and disease [48,49,50] and serve as progenitor cells for specialized fibroblast subtypes and are involved in fibrosis and immunomodulation [51,52,53,54].

Stroma cells fulfilling MSC criteria have been isolated from the human [55] as well as murine [56,57] pancreas. They are mainly found in the perivascular area as well as in the intra- and interlobular stroma tissue, where they surround the glandular structures of the pancreas [55,58,59].

MSCs isolated from human islet fractions (LPI) as a by-product of islet cell transplantation showed typical defining characteristics as MSCs from other sources but exhibited higher expression of nestin and angiogenic markers but a lower expression level of CD146 and a lower adipogenic differentiation potential [55]. MSCs harbor an immunomodulating capacity and in healthy tissue, they support an immunosuppressive environment [60]. This involves the release of soluble factors with anti-inflammatory, anti-oxidative, and anti-apoptotic properties (e. g. IL-6, IL-10, TGF-β, PGE2, CCL2, or VEGF) but also secretion of extracellular vesicles comprising miRNAs, cytokines, growth factors, and cell–cell contact including receptor–ligand interactions (e.g., PD-L1 and PD-L2). To date, there is no clear evidence of the specific relation between MSCs and PSCs.

There are controversial reports on the trans-differentiation of both non-pancreatic and pancreatic MSCs into pancreatic epithelial cells. Cao et al. also report the in vitro differentiation of immortalized MSCs from fetal porcine pancreas into insulin-producing beta cells [61]. In contrast, Seeberger et al. showed that the occurrence of EMT in pancreatic cells is an artifact of in vitro cell culture and does not represent a natural process in pancreatic tissue or tumor development [62]. In our view, despite similarities in surface marker expression [48], pancreatic MSCs and PSCs represent differential cell populations with a differential impact on pancreatic pathobiology.

## 3. Alterations and Differentiation of Stroma in Pancreatic Inflammation

**Figure 3:**
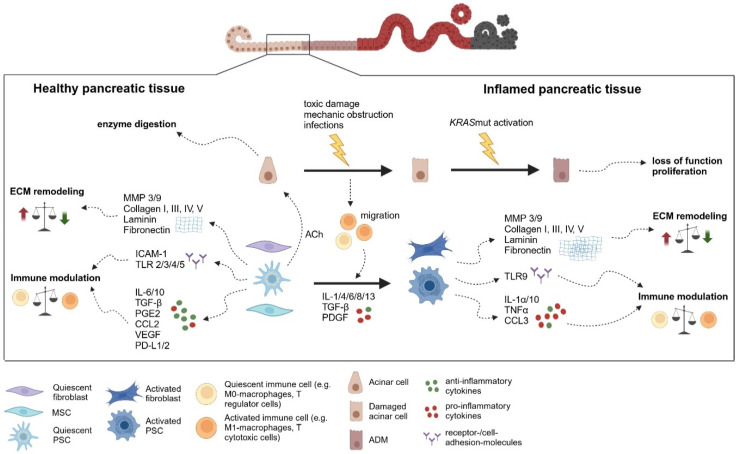
Relevant molecular processes in the pancreatic stroma in chronic pancreatitis as a precursor for PDAC carcinogenesis. The figure illustrates cellular and molecular mechanisms that lead to inflammation in the pancreas. *Healthy pancreatic tissue*: intact acinar cells are responsible for enzyme digestion. Resting fibroblasts, pancreatic stellate cells (PSCs), and multipotent stromal cells (MSCs) ensure functional and immunological homeostasis: the balance between formation and degradation of the extracellular matrix (ECM), including all its components such as collagen, laminin, and fibronectin is maintained. Matrix metalloproteinases (MMPs) regulate the synthesis and degradation of ECM. Anti-inflammatory cytokines, such as IL-6, IL-10, TGF-β, and PGE2, as well as chemotactic factors such as CCL2 and VEGF, promote an immunosuppressive microenvironment. PSCs in particular regulate acinar cell function through acetylcholine (ACh). *Inflamed pancreatic tissue*: toxic damage, mechanical obstruction, and infection lead to cell damage in acinar cells. Activated immune cells migrate into the tissue and promote the differentiation of quiescent mesenchymal cells into activated fibroblasts and PSCs. As a result, collagen and fibronectin are increasingly deposited and MMPs are dysregulated, leading to greater ECM formation than degradation. Pro-inflammatory cytokines such as IL-1α, IL-10, TNFα and CCL3 dominate. TLR9 receptors are activated, which further strengthens the immune response. The consequence is the activation of oncogenic KRAS and increased proliferation of non-functional acinar cells. Created in https://BioRender.com.

Based on their clinical course and pathobiology acute and chronic pancreatitis are distinguished. Acute pancreatitis represents a short-term inflammation of the exocrine pancreas and is characterized primarily by an infiltration of granulocytes accompanied by fluid retention [4]. In mild cases, involving only the pancreas, no long-term sequelae are prominent. Severe cases involving a systemic inflammatory response syndrome can lead to necrosis including changes in the pancreatic stroma [63]. In contrast, chronic pancreatitis reflects a recurrent and persistent inflammatory process either preceded by acute pancreatitis [64]. It eventually results in fibrosis, increased stroma presence, loss of functional epithelia, and metaplasia [65]. During this process, the stroma modifies inflammation and is modified itself by inflammation (Figure 3).

The current literature suggests that the pancreatic stroma has a significant impact on the course of inflammatory responses in the pancreas facilitating carcinogenesis of pancreatic ductal adenocarcinoma (PDAC) [66,67].

### 3.1. Stroma in Acute Pancreatitis

Acute pancreatitis—caused by toxic damage, ductal obstruction, or, in rare cases, infections—represents an acute inflammatory process mostly characterized by the infiltration of hematopoietic cells, particularly granulocytes and fluid retention with subsequent release of pancreatic enzymes into the tissue [68]. Although an activation of PSCs in acute pancreatitis has long be known [69], generally no significant expansion or fibrotic changes in the stroma ensue. In cases of mild injury, the tissue can be rapidly restored by the reactivation of resident progenitor cells that differentiate into new acinar cells, without permanent structural damage and no association with malignant transformation [70].

However, the fact that repeated episodes of acute pancreatitis predispose the development of chronic pancreatitis suggests that pancreatitis represents a dynamic inflammatory process where stroma alterations are present from early on [64,68]. Data on the specific contribution of stroma cells to either the inflammatory process or its containment in acute pancreatitis are sparse. Recent data support the notion that the pancreatic stroma contributes to the inflammatory process in acute pancreatitis. Specifically, resident fibroblasts and PSCs are activated by various inflammatory signals during acute pancreatitis [38]. Based on their known immunosuppressive potential a multitude of studies have explored the therapeutic effects of MSCs or MSC-related products like exosomes in acute pancreatitis [71]. However, it remains speculative whether pancreatic MSCs are decisive for controlling inflammation in acute pancreatitis.

### 3.2. Stroma in Chronic Pancreatitis

Chronic pancreatitis is often associated with alcohol consumption, smoking, and nutritional factors but also hereditary disposition or efferent duct aberrations [72]. In contrast to acute pancreatitis, it is characterized by an increase in stroma ensuing fibrosis [4] and is a predisposing factor for pancreatic cancer [73].

In general, the chronic inflammatory stimulus with persistent immune cell infiltration leads to dedifferentiation, subsequent re-maturation, and also atrophy of acinar cells [68]. Pancreatic stroma cells may directly impact the inflammation, but data are sparse. For instance, PSCs are a source of cytokines like IL-4Ra ligand which promotes macrophage M2 polarization [74]. The chronic inflammatory response is accompanied by alterations in the proportion and characteristics of pancreatic stroma cells, i.e., pancreatic fibroblasts, PSCs, and probably MSCs as exemplified in Figure 3. Specifically, upon chronic pancreatitis, PSCs and probably other pancreatic stroma cells undergo an activation process mainly induced by pro-inflammatory cytokines like IL-1, IL-4, IL-6, IL-8, IL-13, and many others. In the context of chronic pancreatitis this activation is poorly defined but for PSCs mainly involves a myofibroblastic differentiation with induction of marker genes like a-SMA [75]. For instance, in a feed-forward process, PSC-induced M2 macrophages in turn activate PSCs through IL-4-signaling [74]. Accordingly, the inhibition of IL-4 and IL-13 led to a significant reduction in fibrosis in a model of PSC activation [74]. These activated PSCs and presumably pancreatic stroma cells, in general, are also the main source of ECM which is characteristic of the increased stroma proportion and fibrosis in chronic pancreatitis [4]. The activation of PSCs and probably other pancreatic stroma cells upon chronic pancreatic inflammation may result in a phenotype resembling so-called cancer-associated fibroblasts (CAFs). By definition, CAFs represent differentiated stroma cells in the context of malignant lesions. However, a prominent feature of the dominant CAF subtype is their myofibroblastic differentiation and pro-tumorigenic potential. And a CAF-like state may be induced by chronic inflammation. For instance, activation of TLR9—a major regulator of inflammation—in PSCs results in a pro-tumorigenic phenotype of PSCs with expression of various cytokines like IL1α, IL10, TNFα, CCL3, and CCL11 but also ECM regulators like MMP3 and MMP9 [76]. Thus, the increased risk of pancreatic adenocarcinoma (PDAC) development upon chronic pancreatitis may be, at least partly, due to an early differentiation of PSCs and likely also other pancreatic stroma cells to a pro-tumorigenic, CAF-like phenotype (Figure 3).

Only indirect evidence for the role of MSCs in chronic pancreatitis can be drawn from intervention studies. In animal models of chronic pancreatitis, the transplantation of MSCs had immunosuppressive and antifibrotic effects, qualifying them as a target for therapeutic interventions.

### 3.3. Activation of Pancreatic Mesenchymal Cells

As mentioned above, inflammatory stimuli and, in particular, their chronic persistence have an impact on pancreatic stroma cells. Current data suggest that this comprises a myofibrogenic differentiation as in other types of chronic inflammation [77]. Regarding PDAC, it appears intriguing to postulate resident pancreatic fibroblasts, PSCs, and pancreatic MSCs on one and CAFs on the opposite pole of a dynamic and continuous process of inflammation-driven stroma, participating in pancreatic carcinogenesis. However, data on this hypothesis is scarce and requires a better understanding of intracellular signaling and intercellular interaction of pancreatic stroma cells. However, recent data on CAFs, in general, and particularly in PDAC have contributed to this hypothesis.

CAFs form a heterogeneous group of mesenchymal cells which can occur in almost all tissues in the context of carcinoma growth [78]. Recent data describe various subtypes of CAFs; however, the current data are not consistent regarding the distinction of their phenotype and function as well as of their presence in different malignancies. Based on studies in PDAC [79], three main subtypes have been classified so far: myofibroblastic CAFs (myCAFs), inflammatory CAFs (iCAFs), and antigen-presenting CAFs (apCAFs) [80]. These subtypes have also been detected in PDAC [78,81].

According to the current knowledge, in pancreatic tissue, CAFs arise via the activation of resident pancreatic stroma cells or infiltrating mesenchymal cells but the precise cellular origin as a determinator of CAF subtypes needs further confirmation [81]. However, myCAFs specifically are derived from PSCs after injury of the pancreas via stimulation by TGF-β, PDGF, or IL-6 and are localized in close proximity to the cancer cells [82]. Activation of PSCs into myCAFs induces a morphological change from star-shaped to spindle-shaped, accompanied by a loss of the intracellular vitamin A droplet and expression of myofibroblast markers like α-SMA [82]. Functionally, myCAFs increasingly produce ECM, promoting the desmoplastic reaction in PDAC. This fibrotic remodeling not only illustrates the connection to the fibrotic reaction seen in chronic pancreatitis and PDAC but also the likely continuum from activated PSCs in chronic pancreatitis to myCAFs in PDAC.

In contrast, iCAFs are located further away from the cancer cells and are characterized by increased secretion of platelet-derived growth factor-α (PDGF-α) as well as chemokines (CXCL1, CXCL2, CCL2, and CXCL12) and interleukins (IL-1, IL-6, and IL-8). Thus, they appear to be involved in the modulation of immune cells [82]. Further, iCAFs specifically express the hyaluronan synthases HAS1 and HAS2.

The third population, apCAF, was defined primarily based on the expression of MHC-II and CD74 and the ability to present antigens to CD4^+^ T cells in vitro. ApCAFs have been associated with immunomodulation, although it is not fully understood whether they have an immunopromoting or suppressive effect [83].

In a recent work, a CAF classification of four subtypes of proinflammatory CAF (pCAF subtypes A–D) has been suggested [84]. Here, pCAF-A corresponds to myCAF, pCAF-B to iCAF and pCAF-D to apCAF. The additional subgroup of pCAF-C represents a hybrid population between myCAF and iCAF, which both contributes to ECM production and emits inflammatory signals. Moreover, subtypes of CAFs have been assigned based on specific surface markers like ^Zeb1+^CAFs, ^LRRC15+^CAFs, ^CD10+/GPR77+^CAFs, ^CD105+^CAFs ^FAP+/CXCL12+^CAFs, and ^Saa3+^CAFs, but also based on physiological conditions ^Hypoxia+^CAFs, ^Metabolic^CAFs. While all these seem to support PDAC growth, subtypes of ^CD271+/NGFR+^CAFs, ^Gli+^CAFs, or ^Meflin+^CAFs seem to excert a tumor-restraining effect [85,86]. In line with this apparent phenotypic heterogeneity the tumor-promoting or—restraining function of CAF in PDAC is disputed [87,88,89]. Compared to the vast data on CAFs in fully developed PDAC, little is known about their role in early, only inflammation-driven carcinogenesis.

## 4. Role of Inflammation-Driven Stroma at Different Stages and Processes of PDAC Carcinogenesis

**Figure 4:**
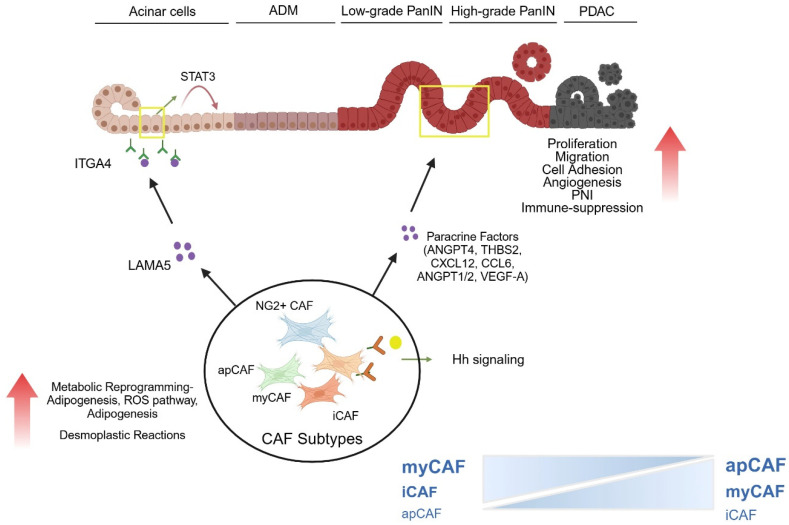
Relevant molecular processes in the pancreatic stroma in early lesions (ADM, PanIN) of PDAC carcinogenesis. Depicted is the development of pancreatic adenocarcinoma (PDAC) and the role of cancer-associated fibroblasts (CAFs) in the tumor microenvironment. The functional heterogeneity of CAFs and their influence on the progression from healthy pancreatic tissue to malignant PDAC via various precursors (ADM, PanIN) are demonstrated. Tumor progression is influenced by signaling pathways, such as STAT3, which contribute to tumor cell activation. The four CAF subtypes (MyCAFs, iCAFs, apCAFs, and NG2^+^ CAFs) are dynamically interconverted by the Hedgehog (Hh) signaling pathway. CAFs secrete laminin-α5 (LAMA5), an integrin-α5 (ITGA4) that is bound by acinar cells. This activates signaling pathways, such as STAT3, which contribute to the progression and persistence of ADM. Furthermore, CAFs influence tumor development in higher precancerous stages through paracrine factors (e.g., ANGPT4, THBS2, and VEGF-A) and promote proliferation, migration, cell adhesion, angiogenesis, and immunosuppression in the tumor environment. Additionally, CAFs contribute to the desmoplastic reaction, which means the fibrotic remodeling of the pancreatic tissue characteristic of PDAC. This leads to metabolic reprogramming, adipogenesis, and oxidative stress. Created in https://BioRender.com.

The relation of pancreatic stroma and fully established PDAC has been explored and reviewed by many studies [90]. In contrast, the role of pancreatic stroma in the carcinogenesis of PDAC and, in particular, at early inflammation-driven stages has been less explored. However, recent data on the interaction of stroma cells with dysplastic pancreatic epithelium as well as pancreatic neuronal and immunoregulatory cells has given some insights into the role of stroma in early stages of PDAC and demonstrates a multitude of changes in pancreatic stroma cells (Figure 4).

### 4.1. Impact of Stroma on ADM Development and ADM—PanIN Transition

Acinar-to-ductal metaplasia (ADM), a key initiating-event in pancreatic carcinogenesis, occurs in response to factors such as cell injury, inflammation, or oncogenic *KRAS* mutations [91,92]. It is well established that *KRAS*-mutant cells interact with the desmoplastic stroma, transforming resident fibroblasts into CAFs [93]. While several mechanisms regulating PDAC progression and neoplastic lesions have been extensively studied [94,95], the microenvironment of precursor lesions remains less understood. Oncogenic *KRAS* mutations induce RAS signaling activity, driving sustained ADM, particularly in the presence of inflammatory or growth signals [96]. This persistent activation is amplified by the induction of inflammatory mediators via NF-κB, Cox2, and STAT3 which recruit immune cells [97,98], and activate key receptors, including GPCRs (for cholecystokinin, PGE2), TLRs (TLR4 for LPS), and EGF/HER receptors (for TGF-β, AREG-amphiregulin) [99]. These signaling feedback loops create a self-sustaining environment that promotes ADM persistence and progression to PanIN.

Recent findings using the *iKras* model, which enables inducible and reversible Kras^G12D^ expression in pancreatic epithelial cells, demonstrate that fibroblast reprogramming precedes ADM formation. One-week post-induction, focal ADM lesions emerged despite an otherwise histologically normal pancreas. Notably, just within three days of Kras^G12D^ activation, untransformed acinar cells were already surrounded by infiltrating macrophages and α-SMA^+^ fibroblasts, highlighting the potential role of activated fibroblasts in ADM initiation [100]. To further investigate the role of reprogrammed fibroblasts in the preinvasive microenvironment, a separate study utilized acinar cells and myCAFs derived from LSL-Kras^G12D/+^; Pdx1Cre (KC) mice compared with wild-type controls. Direct co-culture of pancreatic acinar organoids with myCAFs, as well as indirect co-culture with myCAF-conditioned media, revealed that myCAFs promote ADM. Mechanistically, CAF-secreted laminin α5 (LAMA5) interacts with integrin α4 (ITGA4) on acinar cells, leading to STAT3 activation and acino–ductal transition [101].

### 4.2. Impact of Stroma on Transition from Low Grade to High Grade PanIN

The presence of CAFs and subtypes in PanIN lesions has been described, suggesting a role in early-stage pancreatic carcinogenesis. Single-cell RNA-seq data demonstrate that myCAFs and iCAFs can be observed in premalignant lesions in both murine models and human samples, suggesting that CAF differentiation occurs early during tumorigenesis [102,103].

Recent studies employing a combinatorial approach—including whole transcriptome analysis from FFPE spatial transcriptomics and single-cell sequencing—have enabled the investigation of rare matched low-grade and high-grade PanIN lesions. This has provided key insights into PDAC evolution and cellular phenotypes. For the first time, the presence of all major CAF subtypes (myCAF, iCAF, and apCAF) has been identified in premalignant human PanIN lesions [102]. Furthermore, these studies suggest a gradual decline in CAF-associated inflammatory and epithelial–mesenchymal transition (EMT)-related pathways during the progression from low-grade to high-grade PanINs. Notably, cancer stem cell (CSC) markers have been detected in PanIN lesions [102]. Using a combination of bulk sequencing, proteomics/phosphoproteomics, single-cell sequencing, spatial transcriptomics, and high-resolution cellular imaging on 83 PDAC samples, transitional cell populations, including ADM and PanIN cells, alongside non-transformed acinar, ductal, and PDAC cells were identified. Interestingly, a small population of CD133^+^ iCAFs expressing CSC markers was found within PanIN lesions but not in PDAC. CSCs are known to drive aggressive tumor behavior and are associated with therapy resistance, local recurrence, and metastasis. The presence of CSCs and iCAFs in PanINs suggests that key characteristics linked to therapy resistance in PDAC may develop as early as the PanIN stage [104].

### 4.3. Impact on Metabolism of Precursor Lesions

CAFs play a critical role in PDAC metabolism by supplying essential nutrients to support tumor growth. For instance, CAFs undergo autophagy in response to signals from cancer cells, leading to the secretion of alanine, which can serve as an alternative fuel for tumor cells by feeding into the TCA cycle and supporting the synthesis of nonessential amino acids and lipids [105]. Furthermore, single-cell RNA sequencing has identified a CAF population with activated adipogenesis as marked by ABCA8a expression putatively allowing for lipid transfer to tumor cells to fuel oxidative phosphorylation [106]. However, while the role of CAFs in PDAC metabolism has been extensively studied, their involvement in the metabolic reprogramming of early pancreatic lesions remains underexplored. A recent study examined the transcriptional profiles of fibroblasts isolated from KPC mice at different time points and showed that fibroblasts in KPC mice during early-stage pancreatic carcinogenesis displayed significant upregulation of hallmark metabolic pathways, including ROS signaling, fatty acid metabolism, and adipogenesis [107].

### 4.4. Angiogenesis in the Stroma During Early-Stage Pancreatic Carcinogenesis

Several studies indicate that angiogenic factors are upregulated in the early stages of PanIN. Immunohistochemical analysis of 32 chronic pancreatitis and 38 PDAC patients with PanIN lesions revealed a progressive increase in CEACAM1 and CEACAM5 expression from PanIN-1 to PanIN-3 compared to normal ducts. CEACAMs, members of the GPI-anchored immunoglobulin (Ig) superfamily, are associated with angiogenesis and metastasis [108]. Additionally, other angiogenic factors such as HIF-2α (modulating Wnt signaling) [109], urocortin [110], and neuropeptide Y receptor 2 (Y2R) [111] are upregulated in PanINs, suggesting that angiogenesis is an early event in pancreatic tumorigenesis. However, the specific role of CAFs and stroma cells in mediating angiogenesis or regulating angiogenic factors within the precursor lesion remains less known.

Recent studies identified THBS2 (Thrombospondin-2), a key regulator of cell motility and angiogenesis, as highly expressed in the stroma of PanIN lesions in both KC and KPC mouse models, as well as PDAC patient tumors. THBS2 expression was CAF-specific, first emerging in early PanIN-I and progressively increasing during PDAC progression. Mechanistically, TGF-β1 from pancreatic cancer cells activated CAFs, leading to THBS2 induction via the Smad2/3 pathway. THBS2 in turn promoted cancer cell growth, proliferation, and adhesion by activating MAPK signaling through integrin αvβ3/CD36 binding. However, its direct role in angiogenesis was not examined in this study [112]. In pancreatic cancer, Hedgehog (HH) signaling functions in a paracrine manner, where tumor epithelial cells secrete HH ligands that signal to stromal cells [113]. The HH coreceptors, GAS1, BOC, and CDON, are expressed in fibroblasts from both normal and KC pancreata. In KC mice three weeks after caerulein-induced acute pancreatitis, these coreceptors were enriched in the stroma surrounding PanIN lesions, with further expansion in later stages. To investigate their role, Gas1^−/−^; Boc^−/−^ double-knockout mouse embryonic fibroblasts (MEFs) were generated and treated with SHH-conditioned medium, confirming attenuated HH-signaling in fibroblasts. In an MEF-transplant tumor model, tumor cells were implanted either alone or co-injected with wild-type MEFs, double-knockout Gas1^−/−^; Boc^−/−^ MEFs, or triple-knockout Gas1^−/−^; Boc^−/−^; Cdon^−/−^ MEFs to assess their impact on tumor development. Stromal deletion of Gas1 and Boc reduced HH pathway activation but paradoxically enhanced tumor growth and angiogenesis, leading to increased expression of VEGFα, ANGPT1, and ANGPT2. While the deletion of all three co-receptors (Gas1, Boc, and Cdon) leads to a near-complete loss of HH-signaling, which in turn severely impairs tumorigenesis and angiogenesis. These findings suggest that CAFs regulate early tumor angiogenesis through HH-signaling, and their dysregulation may drive an angiogenic switch that facilitate PanIN progression [114].

### 4.5. Impact on Intrapancreatic Nerves and Precursor–Neural Interactions

Sensory neurons transmitting from the pancreas to the CNS and autonomic neurons including sympathetic, parasympathetic, and enteric nerves form a complex network in the pancreas [115]. In rodent models of acute and chronic pancreatitis, blocking sensory neuron fibers from both the nodose and spinal ganglia has been shown to reduce inflammation [116]. In pancreatic carcinogenesis, sensory neurons establish a reciprocal signaling loop between the pancreas and the CNS, driving spinal and pancreatic inflammation, perineural invasion (PNI), and PanIN formation. Markers of spinal cord inflammation were found to be elevated in mice models at both PanIN and PDAC stages. Additionally, in these mice, acinar-derived cells migrated to the dorsal root ganglion during the PanIN stages, indicating that preneoplastic cells interact with sensory neurons. Sensory neuron ablation in these models prevented PNI, reduced glial activation, and neuronal damage, and significantly delayed PanIN formation [117].

Traditionally, neurogenic invasion (NI) has been viewed as a process in which cancer cells invade nerves to facilitate tumor spread. However, recent findings suggest that Schwann cells within the tumor stroma and not malignant cells themselves, may migrate first during the premalignant phase in human and murine PDAC samples [118]. Schwann cell migration occurs in early PanIN lesions and is correlated with a higher frequency of PanIN in the malignant phase [118]. Tumor-associated chemokines, such as CXCL12, recruit Schwann cells to PanIN lesions via CXCR4 and CXCR7 [119]. The pancreatic neuroectodermal–mesenchymal network includes perilesional pericytes—specialized mural cells originating from Nkx3.3-expressing pancreatic mesenchyme—as well as glial cells, i.e., astrocytes. These components contribute to various functions, such as fibrosis scar after tissue injury, local blood flow regulation by adjusting capillary diameter in response to sympathetic adrenergic input, and support of islet basement membranes through ECM production, including laminin [120]. Recent studies using KrasG12D-mutant mice and optically cleared pancreatic tissue allowed for a 3D visualization of PanIN lesion development following repetitive cerulein injections. These studies revealed the clustering of PanINs in spatially organized aggregates, and upregulation of pericyte (NG2^+^) and glial (GFAP+) markers near PanIN lesions, indicating their activation, and formation of PanIN-islet complexes. Zooming into the PanIN-associated stroma further revealed the presence of NG2^+^ myofibroblast-like cells (α-SMA^+^/NG2^+^) around PanIN lesions. This suggest either a pericyte-to-myofibroblast transition or molecular phenotypic changes in stromal cells in response to preneoplastic lesions [121,122]. Hence, dynamic interactions between epithelial transformation, stromal reprogramming, and neurovascular changes contribute to PanIN progression [122].

### 4.6. Impact on Immune Cell Distribution and Precursor–Immune Cell Interactions

As mentioned above, various CAF subtypes—specifically iCAFs and myCAFs—exhibit distinct functional roles in shaping the tumor microenvironment. Based on current knowledge iCAFs are the key producers of inflammatory cytokines such as IL-6/8/11 and CXCL1/2/12 as well as leukemia inhibitory factor (LIF) [82,83]. In contrast, myCAFs are characterized by their abundant production of ECM components, particularly collagens I, which can act as a physical barrier to immune cells and therapeutic interventions but also potentially restrains preneoplastic lesions from progressing to invasive cancer [87].

Selective depletion of α-SMA^+^ myCAFs during both the PanIN and PDAC stages weakened immune surveillance, thereby facilitating tumor progression. The impact of depletion was more pronounced at the PanIN stage, where a significant reduction in CD45^+^ immune cells including CD3^+^ T cells, CD19^+^ B cells, and CD4^+^Foxp3^−^ effector T cells (Teffs) was observed, along with an increase in immunosuppressive CD4^+^Foxp3^+^ Tregs surrounding PanIN lesions [123]. Hence, the formation of PanIN is associated with the infiltration of immunosuppressive cells while cytotoxic cells remain scarce. In accordance with the known role of CD8^+^-T cells in anti-tumor immunity, their activation at preinvasive stages has been shown to effectively block PanIN progression [124]. Moreover, CD4^+^CD25^+^Foxp3^+^ regulatory T cells (Tregs) progressively accumulate in both mouse and human PanIN and PDAC [124,125]. Given their well-established role in immunosuppression, it was expected that Tregs would actively contribute to immune evasion during early pancreatic carcinogenesis. However, studies using Treg depletion in KC mouse models revealed that Treg depletion failed to relieve immunosuppression and led to accelerated tumor progression. Analysis of single-cell RNA-seq data of mouse PanIN lesions revealed that Tregs serve as key sources of TGFβ1, while both epithelial cells and pancreatic fibroblasts express TGFβ receptors in and around PanINs. The loss of Treg triggers fibroblast reprogramming, probably shifting tumor-restricting α-SMA-high myCAFs into a tumor-promoting inflammatory phenotype, characterized by increased chemokine secretion (*Ccl3*, *Ccl6*, and *Ccl8*) that attracts suppressive myeloid cells and elevated expression of immune-suppressive genes like Arg1 and CD274 (PD-L1). These findings underscore the importance of fibroblast–Treg interactions in the early stages of carcinogenesis. Notably, myCAFs are rare in low-grade dysplastic-IPMNs, but become highly represented in high-grade dysplastic-IPMNs, suggesting a progressive activation of fibroblasts into the myCAF phenotype during preinvasive lesion development. iCAFs are exclusively found in PDAC clusters but are absent in non-invasive IPMNs [126]. Several studies have linked iCAF-derived factors to immune suppression [82,127], indicating that while myCAFs may contribute to fibrosis and immune evasion due in early tumorigenesis, iCAFs later drive immunosuppression through cytokine and chemokine signaling. However, further validation is needed to confirm this transition and its functional implications in PDAC progression. In conclusion, stromal heterogeneity dynamically evolves throughout multistep carcinogenesis, progressively shaping the microenvironment to facilitate the transition from preinvasive IPMN to invasive PDAC. Another CAF subtype, apCAF, has been found adjacent to PanIN lesions, co-localizing with CD4^+^ T cells, suggesting early-stage precursor–immune cell interactions. As their key feature apCAFs express MHC class II molecules, allowing them to present antigens to CD4^+^ T cells. However, unlike conventional antigen-presenting cells, apCAFs lack key costimulatory signals required for full T cell activation and proliferation. Moreover, a negative correlation has been observed between apCAF abundance and the effector CD8^+^ T-cell/Treg ratio in human PDAC. Instead of promoting anti-tumor immunity, MHC-II on apCAFs may function as a decoy receptor, leading to CD4^+^ T cell dysfunction or inducing their conversion into Treg, thereby reinforcing immune suppression in the tumor microenvironment [83]. While the presence of apCAFs in PanIN lesions suggests a potential immunosuppressive role in early carcinogenesis, further validation is required to fully elucidate their function and therapeutic potential.

## 5. Current Translation into the Clinical Setting and Outlook

**Figure 5:**
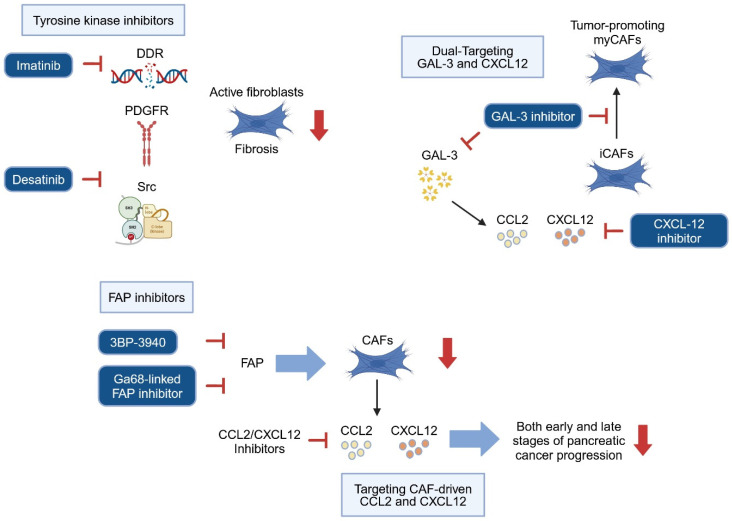
Therapeutic strategies to target stroma in inflammation-driven pancreatic carcinogenesis. Tyrosine kinase inhibitors such as Imatinib and Dasatinib inhibit PDGFR and Src signaling, reducing fibroblast activation and fibrosis. FAP inhibitors, including 3BP-3940 and Ga68-linked FAP inhibitors, selectively deplete tumor-promoting fibroblasts. Strategies targeting CAF-driven chemokines like CCL2 and CXCL12 aim to disrupt tumor-stromal interactions at both early and late stages of pancreatic cancer progression. Additionally, dual inhibition of Galectin-3 (GAL-3) and CXCL12 is proposed to modulate iCAFs and tumor-promoting myCAFs. These interventions have potential for reprogramming the tumor microenvironment and improving therapeutic efficacy in pancreatic cancer. Inhibition symbols are colored red. Created in https://BioRender.com.

Recent scientific evidence highlights that identifying and selectively targeting tumor-promoting CAFs, as well as reprogramming them into tumor-restraining CAFs, are promising strategies for advancing pancreatic cancer therapy [128]. Targeting specific CAF subtypes as well as reprogramming and deactivating fibroblasts at early stages of pancreatic carcinogenesis holds potential for significantly enhancing treatment efficacy. Such early interventions may pave the way for more effective therapeutic approaches, potentially disrupting the progression of pancreatic cancer precursors in clinical settings (Figure 5).

### 5.1. Targeting Stroma Cells to Prevent Precursor Progression

As previously mentioned, activated fibroblasts upregulate several proteins including α-SMA, DDR2, desmin, FAP, vimentin, and PDGF receptors, and depletion of these factors could be considered as a therapeutic strategy. However, in preclinical pancreatic cancer mouse models, the deletion of α-SMA-positive cells promotes the development of hypoxic, invasive, undifferentiated tumors, and decreases animal survival [87]. Especially in the precursor stage, active fibroblasts could play important roles for tissue regeneration, thereby depletion of fibroblasts may enhance tissue injury leading to pancreatic cancer precursor progression. Interestingly, pharmacological targeting of DDR1 and DDR2 by Imatinib, which is also an inhibitor for Bcr-Abl, c-Kit, and PDGFR [129,130], attenuates pancreatic fibrosis by inhibiting activation of PSCs via TGFβ/SMAD signaling in a cerulein-induced chronic pancreatitis mouse model [34]. Another tyrosine kinase inhibitor Dasatinib also attenuates inflammation and fibrosis by inhibiting various tyrosine kinases such as PDGFR and Src in a cerulein-induced chronic pancreatitis mouse model [131]. Additionally, the PARP-inhibitor Olaparib has shown beneficial effects in the same chronic pancreatitis model, reducing tissue injury, inflammation markers (*Il1b*, *Tnfa*, and *Il6*) and fibrosis markers (*Tgf1b*, α-SMA and *Col1a1*), suggesting direct or indirect fibroblast deactivation through PARP-inhibition [132]. These findings indicate that inhibiting fibroblast activation may help curb precursor lesion progression. However, further clarification is needed to determine whether these anti-fibrosis effects stem from the direct targeting of activated fibroblasts or from broader effects on tissue injury and inflammation.

Another promising target for preventing precursor progression is FAP, as deletion of FAP-positive CAFs has been associated with increased survival in contrast to the depletion of α-SMA-positive CAF in a PDAC mouse model [133]. Recently, novel selective FAP inhibitors have been developed and paired with radiotracers for use in positron emission tomography/computer tomography (PET/CT) [134] and have been tested in patients with IPMN and pancreatic cancer [135,136]. Interestingly, cumulative FAP expression in both CAFs and epithelial cells is upregulated at the stage of high-grade dysplasia and persists throughout the progression of pancreatic cancer [137]. These findings suggest that FAP could serve as a valuable target for both CAFs and neoplastic cells. Inhibiting FAP combined with radiotracer technology may ultimately aid in the early diagnosis and treatment of pancreatic cancer precursors.

### 5.2. Targeting Paracrine Communication Between Stroma and Precancerous Cells

FAP-positive CAFs are key sources of the chemokine CCL2, which plays a central role in recruiting monocyte-derived suppressor cells (MDSCs) and promoting tumor progression. This recruitment function is significantly reduced in conventional *Ccr2* knockout mice, indicating the importance of CCL2 in the tumor-supporting microenvironment created by FAP-positive CAFs [138]. CCL2 production is not limited to FAP-positive CAFs—TGF-β, for example, stimulates pancreatic cancer cells to secrete CCL2 and recruit macrophages [139]. However, targeting the CCL2/CCR2 signaling axis remains a promising therapeutic approach. In experimental models of acute pancreatitis, treatment with the CCL2 synthesis blocker Bindarit reduced macrophage infiltration and alleviated pancreatic injury and inflammation, suggesting its potential utility in early pancreatic carcinogenesis [140,141]. Additionally, FAP-positive CAFs express CXCL12 [142], a chemokine that interacts with receptors CXCR4 and CXCR7 [143], both commonly co-expressed in pancreatic cancer tissues [144]. Studies have shown that in early pancreatic cancer cell lines, knockdown of CXCR7 inhibits CXCL12-driven proliferation, while in more advanced cell lines, silencing CXCR4 effectively reduces proliferation [144]. This suggests that targeting CAF-driven CXCL12 signaling could offer therapeutic benefits across both early and late stages of pancreatic cancer progression.

Targeting the reciprocal communication between CAFs and tumor cells, wherein paracrine factors from precancerous cells induce CAFs to produce additional tumor-promoting signals, represents another promising strategy. Galectin-3 (GAL3), a beta-galactoside-binding lectin, is associated with chronic inflammation, fibrosis, and cancer [145] and has been shown to activate pancreatic stellate cells (PSCs) while stimulating IL-8 and CCL2 production [146]. In a pancreatic cancer mouse model, genetic deletion of Galectin-3 (*Pdx1-Cre*; *LSL-Kras^G12D/+^*; *Trp53^lox/lox^*; *Lgals3^−/−^*) effectively inhibited tumor progression [147]. Interestingly, this deletion also preserved an early-stage CAF subtype composition, with a high proportion of iCAFs expressing elevated levels of CXCL12 and a reduced myCAF subtype, contrasting with the CAF composition observed in late-stage pancreatic cancer, where myCAFs predominate [147]. This CAF profile in early-stage pancreatic cancer suggests that targeting both Galectin-3 and CXCL12 could reinforce therapeutic efficacy, especially when tailored to tumor stage [147].

In summary, selective targeting of chemokine pathways like CCL2/CCR2 and CXCL12/CXCR4-CXCR7, alongside key mediators such as Galectin-3, presents a multifaceted approach to intercepting CAF-driven tumorigenesis. These strategies hold potential to disrupt the tumor-promoting microenvironment in early pancreatic carcinogenesis, laying the groundwork for more effective and stage-specific therapeutic interventions.

## 6. Conclusions

PDAC remains one of the most lethal malignancies largely due to its complex and highly desmoplastic tumor microenvironment. The emerging evidence suggests that pancreatic stroma cells, like PSCs, MSCs, and CAFs, play a pivotal role in early-stage pancreatic carcinogenesis, from ADM and PanIN progression to invasive PDAC. The interactions between these and immune cells, neural components, and pre-invasive epithelial lesions collectively create an environment that fosters tumor progression while simultaneously suppressing antitumor immunity. Notably, pancreatic stroma cell heterogeneity presents a significant challenge, with subpopulations exerting either tumor-promoting or tumor-restraining functions. Understanding the precise mechanisms of pancreatic stroma cell activation and differentiation as well as function remains essential for developing effective therapeutic strategies. Further, recent findings highlight the metabolic reprogramming of fibroblasts as well as the intricate crosstalk between fibroblasts and neural components in pancreatic carcinogenesis. Despite substantial progress in understanding the role of inflammation-driven stromal remodeling, targeting pancreatic stroma remains a double-edged sword. Strategies such as reprogramming tumor-supportive stroma cells into tumor-restraining phenotypes or modulating key paracrine interactions hold promise for future therapeutic interventions. Moreover, emerging diagnostic approaches may offer novel opportunities for early detection and intervention in pancreatic cancer precursors. In conclusion, the interplay between stroma and the epithelium undergoing malignant transformation represents a critical yet underexplored dimension of early-stage pancreatic carcinogenesis. Future studies should focus on refining our understanding of pancreatic stroma cell plasticity, identifying actionable targets for therapeutic intervention, and integrating stromal-targeting strategies into multimodal treatment approaches. In doing so, we may enhance early detection and improve outcomes for patients with PDAC.
